# Complaints of Sleep Disturbances Are Associated with Cardiovascular Disease: Results from the Gutenberg Health Study

**DOI:** 10.1371/journal.pone.0104324

**Published:** 2014-08-05

**Authors:** Matthias Michal, Jörg Wiltink, Yvonne Kirschner, Astrid Schneider, Philipp S. Wild, Thomas Münzel, Maria Blettner, Andreas Schulz, Karl Lackner, Norbert Pfeiffer, Stefan Blankenberg, Regine Tschan, Inka Tuin, Manfred E. Beutel

**Affiliations:** 1 Department of Psychosomatic Medicine and Psychotherapy, University Medical Center Mainz, Mainz, Germany; 2 Department of Medicine II, University Medical Center of the Johannes Gutenberg-University Mainz, Mainz, Germany; 3 Institute of Medical Biostatistics, Epidemiology & Informatics, University Medical Center of the Johannes Gutenberg-University Mainz, Mainz, Germany; 4 Department of General and Interventional Cardiology, University Medical Center Hamburg-Eppendorf, Hamburg, Germany; 5 Institute of Clinical Chemistry and Laboratory Medicine, University Medical Center of the Johannes Gutenberg-University Mainz, Mainz, Germany; 6 Center for Thrombosis and Hemostasis, University Medical Center Mainz, Johannes Gutenberg University Mainz, Mainz, Germany; 7 Department of Ophthalmology, University Medical Center Mainz, Mainz, Germany; 8 Preventive Cardiology and Preventive Medicine, Department of Medicine II, University Medical Center of the Johannes Gutenberg-University Mainz, Mainz, Germany; 9 German Center for Cardiovascular Research (DZHK), partner site Rhine-Main, Germany; University of Bologna, Italy

## Abstract

**Background:**

Despite their high prevalence, sleep disorders often remain unrecognized and untreated because of barriers to assessment and management. The aims of the present study were to examine associations of complaints of sleep disturbances with cardiovascular disease, related risk factors, and inflammation in the community and to determine the contribution of sleep disturbances to self-perceived physical health.

**Method:**

The sample consists of n = 10.000 participants, aged 35 to 74 years of a population based community sample in Germany. Cross-sectional associations of complaints of sleep disturbances with cardiovascular risk factors and disease, biomarkers of inflammation, depression, anxiety, and physical health status were analyzed.

**Results:**

19% of our sample endorsed clinically significant sleep disturbances. In the unadjusted analyses severity of sleep disturbances increased with female sex, low socioeconomic status, living without a partnership, cardiovascular disease, depression, anxiety, poor physical health, increased levels of C-reactive protein and fibrinogen. After multivariate adjustment robust associations with coronary heart disease, myocardial infarction and dyslipidemia remained. Complaints of sleep disturbances were strong and independent contributors to self-perceived poor physical health beyond depression, anxiety and medical disease burden.

**Conclusions:**

Given the high prevalence of complaints of sleep disturbances and their strong impact on health status, increased efforts should be undertaken for their identification and treatment.

## Introduction

Impairment of sleep, such as difficulties falling or staying asleep or sleeping too much belong to the most prevalent health complaints of primary care patients and in the community. According to a recent review, about 25% of adults are dissatisfied with their sleep, 10–15% report that their sleep disturbances are associated with negative daytime consequences (e.g. fatigue, low energy), and 6–10% meet criteria for insomnia [1]. Sleep complaints increase with age and they are twice as prevalent in women compared to men [1]. Sleep disturbances are associated with a high rate of medical and mental disorders. The 2002 National Health Interview Survey revealed a 12-month prevalence rate of insomnia or trouble sleeping of 17.4% [2]. Strong positive association between sleep disturbances and common medical conditions were found: 15% increase of obesity, 32% increase of hypertension, 124% increase of congestive heart failure and a 5.64 fold increased likelihood of anxiety or depression. Another recent community-based sample of 3282 men and women aged 18 to 65 years reported a 21.4% prevalence for insomnia [3]. Persons with medical diseases had a 2.2 fold higher likelihood for insomnia as compared to healthy persons. Specifically, the odds ratios (OR) of insomnia were higher in people with heart disease OR 1.6, hypertension OR 1.5, and diabetes OR 1.4 [3]. The prevalence of insomnia increased with the number of medical disorders. Important to note, the associations of insomnia with medical diseases were independent from the findings in polysomnographic measurements [3].

Inflammatory processes are assumed to represent an important biological mechanism linking poor sleep to cardiovascular disease [4], [5]. Although experimental studies clearly indicated that sleep deprivation and insomnia can increase inflammatory processes, the relationships of sleep with inflammation remain far from being settled [4]. A large community based study found no consistent association of sleep disturbance with the inflammatory marker of C-reactive protein [4]. However, poorer self-reported sleep quality was associated with C-reactive protein and fibrinogen levels in a claim sample of n = 340 individuals undergoing in-home polysomnographic monitoring [6]. In a population based sample of n = 188 persons (aged 52 to 70 years) a trend toward higher C-reactive protein was found in individuals who need longer than 30 minutes to fall asleep [7].

Despite their significant burden for the patient and the health care system, sleep disturbances often remain unrecognized and untreated because of barriers to assessment and management. Patients and physicians do not regard sleep disturbances as a disorder that needs to be treated [8]. Therefore a recent study examined, whether a single question could be used as an adequate screener for identifying complaints of sleep disturbances [9]. It was found that this sleep disturbance item from the depression module of the Patient Health Questionnaire showed promise as a screener for sleep problems in primary care.

Therefore the aims of the present study were, 1) to examine associations of complaints of sleep disturbances with cardiovascular disease, related risk factors, and inflammation in the community and 2) to determine the contribution of complaints sleep disturbances to self-perceived physical health.

## Methods

### Ethics statement

Prior to enrolment participants signed written, informed consent. The study has been approved by the local Ethics Committee (Landesaerztekammer Rheinland-Pfalz, 837.020.07).

### Study sample

We investigated cross-sectional data of the first n = 10.000 participants enrolled in the Gutenberg Health Study (GHS) from April 2007 to October 2008 [10]–[13]. The GHS is a population-based, prospective, observational single-center cohort study in the Rhein-Main-Region in western Mid-Germany [13]. The sample was drawn randomly from the local registry in the city of Mainz and the district of Mainz-Bingen. The sample was stratified 1∶1 for gender and residence and in equal strata for decades of age. Inclusion criteria were age 35 to 74 years and written informed consent. Persons with insufficient knowledge of German language, or physical and mental inability to participate were excluded. The current response rate was 55.8%.

### Measures

Sleep disturbances were assessed with the corresponding item #3 of the depression module of the Patient Health Questionnaire (PHQ-9) [14]: “trouble falling or staying asleep, or sleeping too much“. Respondents rated severity of sleep disturbances on a 4 point Likert scale: “Over the last 2 weeks, how often have you been bothered by any of the following problems?” Not at all ( = 0), several days ( = 1), more than half the days ( = 2), nearly every day ( = 3). In the previous validation study the sleep disturbance item correlated strongly with the Insomnia Severity Index (r = 0.75) [9].A cut-off score of 1 was recommended for the screening for sleep problems in primary care. The cut-off score of 1 yielded a sensitivity of 82.5% and specificity of 84.5% [9]. Clinically relevant sleep disorders were defined by sleep disturbances at least more than half the days over the last two weeks.

Anxiety was measured with the 2-item version of the GAD-7 [15]. A cut-off score of 3 or more detects current generalized anxiety disorder (GAD) with a sensitivity of 86% and a specificity of 83%, and it identifies any current anxiety disorder (GAD, panic disorder, social phobia, post-traumatic stress disorder) with a sensitivity of 65% and specificity of 88% [16]. Depression was measured with the two-item depression module of the Patient Health Questionnaire (PHQ-2). A cut-off score of 3 or more yielded a sensitivity of 79% and a specificity of 86% for any depressive disorder [15].

2005). The self-perceived physical health status was assessed by the question “How would you describe your current physical health status?” (“very well  = 1”, “well  = 2”, “less well  = 3”, “badly  = 4”). A binary variable impaired physical health status was denoted by recoding either “less well” or “badly” as impaired physical health status.

### Computer-assisted Personal Interview

Medical history was assessed during the computer-assisted personal interview (e.g. medical history of coronary heart disease, myocardial infarction, stroke etc.). Cardiovascular risk factors were defined as follows: Smoking was dichotomized into non-smokers (never smoker and ex-smoker) and current smokers (occasional smoker, i.e. <1 cigarette/day, and smoker, i.e. >1 cigarette/day). Unhealthy alcohol intake was defined as habitual alcohol intake of >20 gram/day for men and >10 gram/day for women. Obesity was defined as a body-mass index ≥30 kg/m^2^. Diabetes was defined in individuals with a definite diagnosis of diabetes by a physician or a blood glucose level of ≥126mg/dl in the baseline examination after an overnight fast of at least 8 hours or a blood glucose level of ≥200mg/dl after a fasting period <8 hours. Dyslipidemia was defined as a definite diagnosis of dyslipidemia by a physician or an LDL/HDL-ratio of >3.5. Hypertension was diagnosed, if antihypertensive drugs were taken, or mean systolic blood pressure was ≥140 mmHg (diastolic blood pressure ≥90 mmHg) in the 2^nd^ and 3^rd^ standardized measurement after 8 and 11 minutes of rest. A positive family history of myocardial infarction (FH-MI) was defined as at least one myocardial infarction in a female first-degree relative of <65 years or a male first-degree relative of <60 years.

The socioeconomic status (SES) was defined according to Lampert's and Kroll's scores of SES with a range from 3 to 27 (3 indicates the lowest SES and 27 the highest SES) [17].

### Laboratory analysis

Serum lipid levels (total cholesterol, triglycerides, and high-density lipoprotein cholesterol), plasma levels of C-reactive protein (CRP), fibrinogen and albumin levels were measured immediately after blood withdrawal by routine methods; low-density lipoprotein cholesterol was calculated by the Friedewald formula. All other measurements were determined in plasma or serum stored immediately after blood withdrawal and centrifugation at −80°C until analysis. The measurements were done in a blinded fashion in a single batch. C-reactive protein, fibrinogen and albumin were used as markers of inflammation. C-reactive protein was dichotomized into with a cut-off of ≥3 mg/dl (according to other studies, e.g. [18]).

### Statistical analysis

Variables were reported as numbers/percentages, means (±1.96 fold standard deviation) or medians (and interquartile ranges (25^th^/75^th^)) as appropriate. The sample characteristics were displayed stratified for severity of sleep disturbances. Differences in the levels of continuous variables and distribution of categorical variables were tested by the Jonckheere-Terpstra-Test and Cochran-Armitage Trend Test, respectively. Associations of sleep disturbances with cardiovascular disease, cardiovascular risk factors etc. were analyzed by logistic or linear regression analyses. In order to be able to describe dose-dependent associations of sleep disturbance with the dependent variables we determined “not being bothered by sleep disturbances over the last two weeks  = 0” as the reference. Two models of multivariable adjustment were applied (model 1: age and sex; model 2: age, sex, SES, partnership, depression according to PHQ-2≥3, anxiety GAD-2≥3, current smoking, unhealthy alcohol intake, and obesity.

All reported p-values correspond to 2-tailed tests. As this is an exploratory study, no adjustments for multiple testing have been done. P-values are given for descriptive reasons only. Due to the large number of tests, p-values should be interpreted with caution and in connection with effect estimates. Statistical analyses were performed using SAS for Windows 9.2 TS Level 1M0 (SAS Institute Inc.) Cary, NC, USA and IBM SPSS Statistics 20.

## Results

Table 1 characterizes the study sample stratified for severity of sleep disturbances. The majority of the participants were bothered by sleep disturbances at least several days over the last two weeks (64.6%), 9.7% were bothered more than half the days and 9.3% nearly every day. Concerning sociodemographics, increasing severity of sleep disturbances was associated with female sex, lower SES and lower likelihood of living in a partnership. Interestingly, sleep disturbances increased with age in women, but not in men. Sleep disturbances were associated with cardiovascular disease: Increasing severity of sleep disturbances was associated with a higher occurrence of coronary heart disease, myocardial infarction, peripheral arterial disease, dyslipidemia, obesity, clinically significant anxiety and depression as well as a poor physical health status, elevated levels of C-reactive protein and of fibrinogen.

**Table 1:** Sample stratified by severity of sleep disturbances.

	Over the last 2 weeks, how often have you been bothered by any of the following problems? Trouble falling or staying asleep, or sleeping too much				Test of trend *
	Not at all	several days	more than half the days	nearly every day	
	(0)	(1)	(2)	(3)	
	n = 3454	n = 4448	n = 947	n = 902	
	35.4%	45.6%	9.7%	9.3%	
Female % (n)	41.5 (1435)	51.8 (2306)	56.1 (531)	60.8 (548)	<0.0001
Age ♂, mean (age-range)	55.5 (35–74)	55.6 (35–74)	56.2 (35–74)	56.3 (35–74)	0.18
Age ♀, mean (age-range)	53.8 (35–74)	55.5 (35–74)	55.8 (35–74)	56.5 (35–74)	<0.0001
SES (3-21), mean (±1.96 SD)	13.1 (4.4∼22.0)	12.9 (4.3∼21.5)	12.4 (3.9∼20.9)	11.8 (3.2∼20.3)	<0.0001
Partnership, yes, % (n)	84.3 (2913)	82.1 (3652)	78.7 (744)	73.5 (663)	<0.0001
Medical history/somatic health					
Coronary heart disease, % (n)	3.7 (127)	4.0 (176)	5.6 (52)	5.8 (51)	0.0013
Myocardial infarction, % (n)	2.9 (100)	2.4 (106)	4.1 (39)	4.3 (38)	0.017
Stroke, % (n)	2.1 (71)	1.8 (78)	2.1 (20)	1.8 (16)	0.066
Peripheral arterial disease, % (n)	3.2 (108)	3.4 (149)	3.7 (34)	5.1 (45)	0.012
Heart Failure, % (n)	1.0 (35)	1.2 (55)	1.9 (18)	2.2 (20)	0.0015
Atrial Fibrillation, % (n)	2.3 (78)	2.8 (122)	2.9 (27)	3.4 (30)	0.051
Hypertension, % (n)	50.1 (1731)	52.0 (2311)	52.9 (500)	53.6 (483)	0.026
Dyslipidemia, % (n)	28.1 (968)	28.2 (1251)	29.3 (277)	37.8 (340)	<0.0001
Diabetes, % (n)	7.7 (266)	6.5 (290)	8.9 (84)	8.5 (77)	0.31
Smoking (current),% (n)	19.7 (678)	18.5 (819)	20.6 (194)	19.9 (179)	0.79
Obesity, % (n)	25.3 (872)	23.9 (1064)	26.6 (252)	30.6 (276)	0.0046
Unhealthy alcohol intake, % (n)	27.2 (938)	28.5 (1264)	28.3 (267)	26.8 (241)	0.88
Poor physical health status, % (n)	12.9 (444)	18.9 (839)	33.7 (319)	42.9 (387)	<0.0001
Mental distress					
Generalized Anxiety, (GAD-2≥3), % (n)	1.9 (66)	4.5 (200)	15.0 (141)	24.1 (216)	<0.0001
Depression, PHQ-2 ≥3, % (n)	1.1 (37)	3.1 (139)	18.4 (174)	41.4 (373)	<0.0001
Inflammation					
CRP ≥ 3 mg/dl (yes vs. no) †	21.0 (571)	21.5 (745)	23.2 (167)	27.6 (178)	0.0008
Albumin, mg/l, mean ± 1.96 SD †	42.5 (36.7∼48.3)	42.5 (36.6∼48.5)	42.5 (36.2∼48.7)	42.1 (36.04∼48.2)	0.167
Fibrinogen, mg/dl †	317 (276/366)	322 (281/370)	324 (283/371)	328 (286/385)	0.0001

† Subjects with a self-reported influenza infection, common cold or other inflammatory diseases during the last week before examination or CRP≥10 mg/dl were excluded.

* Continuous variables Jonckheere-Terpstra-Test, categorical variables Cochran-Armitage Trend Test, Weighted prevalence rates for the region Mainz-Bingen: 0 = 36.06%, 1 = 45.5%, 2 = 9.6%, 3 = 8.9%, Missing data: N = 249.

In the next step associations of the severity of sleep disturbances with cardiovascular disease, cardiovascular risk factors, health status and inflammation were analyzed by multivariate regression models. In the first model, adjusted for age and sex, severity of sleep disturbances was associated with CHD, myocardial infarction, heart failure, diabetes, dyslipidemia, and elevated C-reactive protein (Table 2). Additional adjustment for SES, partnership, clinically significant depression and anxiety, current smoking, unhealthy alcohol intake, and obesity did not change the associations of severity of sleep disturbances with CHD, MI or dyslipidemia (Table 3). However, the associations of sleep disturbances with heart failure, diabetes, elevated CRP and fibrinogen disappeared.

**Table 2:** Association of the severity of sleep disturbances with cardiovascular disease, metabolic disorders and inflammation.

	Model 1	
*CHD [N = 9604]	p-value <0.001	
	1 vs. 0	1.21 [0.95, 1.54]
	2 vs. 0	1.79 [1.27, 2.53]
	3 vs. 0	1.98 [1.40, 2.81]
*MI [N = 9706]	p-value <0.001	
	1 vs. 0	0.89 [0.68, 1.19]
	2 vs. 0	1.63 [1.103, 2.401]
	3 vs. 0	1.78 [1.203, 2.65]
*Stroke [N = 9704]	p-value: 0.842	
	1 vs. 0	0.88 [0.64, 1.23]
	2 vs. 0	1.05 [0.63, 1.75]
	3 vs. 0	0.91 [0.52, 1.58]
*PAD [N = 9650]	p-value: 0.079	
	1 vs. 0	1.059 [0.82, 1.37]
	2 vs. 0	1.13 [0.76, 1.67]
	3 vs. 0	1.59 [1.11–2.29]
*Heart Failure [N = 9743]	p-value: 0.028	
	1 vs. 0	1.203 [0.78, 1.85]
	2 vs. 0	1.804 [1.01, 3.22]
	3 vs. 0	2.14 [1.01, 3.22]
*Atrial Fibrillation [N = 9647]	p-value: 0.076	
	1 vs. 0	1.32 [0.99, 1.78]
	2 vs. 0	1.37 [0.87, 2.15]
	3 vs. 0	1.71 [1.103, 2.65]
*Hypertension [N = 9748]	p-value: 0.417	
	1 vs. 0	1.069 [0.97, 1.18]
	2 vs. 0	1.096 [0.94, 1.28]
	3 vs. 0	1.108 [0.94, 1.30]
*Diabetes [N = 9741]	p-value: 0.017	
	1 vs. 0	0.86 [0.72, 1.03]
	2 vs. 0	1.22 [0.93, 1.59]
	3 vs. 0	1.19 [0.907, 1.57]
*Dyslipidemia [N = 9731]	p-value <0.001	
	1 vs. 0	1.068 [0.97, 1.18]
	2 vs. 0	1.16 [0.98, 1.18]
	3 vs. 0	1.77 [1.51, 2.072]
*,†CRP ≥ 3 mg/dl [yes vs. no] [N = 7552]	p-value = 0.024	
	1 vs. 0	0.98 [0.87, 1.11]
	2 vs. 0	1.062 [0.87, 1.295]
	3 vs. 0	1.32 [1.08, 1.61]
‡,†Albumin - mg/l [N = 7551]	p-value: 0.056	
	1 vs. 0	0.097 [−0.054, 0.25]
	2 vs. 0	0.057 [−0.19, 0.30]
	3 vs. 0	−0.25 [−0.505, 0.01]
‡,†Fibrinogen, mg/dl [N = 7493]	p-value <0.074	
	1 vs. 0	0.88 [−2.64, 4.41]
	2 vs. 0	2.86 [−2.89, 8.62]
	3 vs. 0	7.85 [1.79, 13.92]

* =  logistics regression: OR [95% CI].

† = Subjects with a self-reported influenza infection, common cold or other inflammatory diseases during the last week before examination or CRP≥10 mg/dl were excluded.

Model 1: age, sex.

‡ =  generalized linear model (GLM): beta [95%CI].

**Table 3:** Association of the severity of sleep disturbances with cardiovascular disease, metabolic disorders and inflammation.

	Model 2	
*CHD [N = 9376]	p-value: 0.019	
	1 vs. 0	1.22 [0.96, 1.56]
	2 vs. 0	1.59 [1.105, 2.28]
	3 vs. 0	1.64 [1.13, 2.39]
*MI [N = 9467]	p-value: 0.006	
	1 vs. 0	0.95 [0.71, 1.28]
	2 vs. 0	1.57 [1.044, 2.36]
	3 vs. 0	1.73 [1.13, 2.63]
*Stroke [N = 9464]	p-value: 0.79	
	1 vs. 0	0.87 [0.63, 1.22]
	2 vs. 0	0.904 [0.53, 1.55]
	3 vs. 0	0.77 [0.43, 1.396]
*PAD [N = 9411]	p-value: 0.34	
	1 vs. 0	1.067 [0.82, 1.39]
	2 vs. 0	1.067 [0.71, 1.61]
	3 vs. 0	1.425 [0.97, 2.101]
*Heart Failure [N = 9503]	p-value: 0.34	
	1 vs. 0	1.23 [0.79, 1.92]
	2 vs. 0	1.56 [0.91, 3.00]
	3 vs. 0	1.55 [0.83, 2.92]
*Atrial Fibrillation [N = 9411]	p-value: 0.31	
	1 vs. 0	1.26 [0.93, 1.69]
	2 vs. 0	1.36 [0.86, 2.16]
	3 vs. 0	1.43 [0.89, 2.31]
*Hypertension [N = 9502]	p-value: 0.54	
	1 vs. 0	1.071 [0.97, 1.19]
	2 vs. 0	1.087 [0.92, 1.28]
	3 vs. 0	1.019 [0.86, 1.21]
*Diabetes [N = 9499]	p-value: 0.52	
	1 vs. 0	0.89 [0.74, 1.074]
	2 vs. 0	1.058 [0.79, 1.41]
	3 vs. 0	0.98 [0.72, 1.33]
*Dyslipidemia [N = 9488]	p-value <0.001	
	1 vs. 0	1.075 [0.969, 1.19]
	2 vs. 0	1.13 [0.95, 1.33]
	3 vs. 0	1.697 [1.43, 2.013]
*,†CRP ≥3 mg/dl [yes vs. no] [N = 7370]	p-value: 0.52	
	1 vs. 0	0.999 [0.88, 1.14]
	2 vs. 0	0.997 [0.806, 1.23]
	3 vs. 0	1.17 [0.94, 1.45]
‡,†Albumin, mg/l [N = 7369]	p-value: 0.41	
	1 vs. 0	0.071 [−0.079, 0.22]
	2 vs. 0	0.095 [−0.15, 0.35]
	3 vs. 0	−0.12 [−0.39, 0.15]
‡,†Fibrinogen, mg/dl [N = 7311]	p-value: 0.50	
	1 vs. 0	1.31 [−2.11, 4.73]
	2 vs. 0	1.49 [−4.17, 7.16]
	3 vs. 0	4.72 [−1.41, 10.85]

* =  logistic regression: OR [95% CI].

† = Subjects with a self-reported influenza infection, common cold or other inflammatory diseases during the last week before examination or CRP≥10 mg/dl were excluded.

‡ =  generalized linear model (GLM): beta [95%CI].

Model 2, adjusted for age, sex, SES, partnership, depression according to PHQ-2≥3, anxiety GAD-2 ≥3, current smoking, unhealthy alcohol intake, obesity.

Severity of sleep disturbances was strongly associated with poor physical health even after multivariate adjustment (Table 4).

**Table 4:** Association of sleep disturbances with self rated poor physical health.

		Model 1 [N = 9742]	Model 2 [N = 9124]
Poor physical health status		p-value ≤0.001	p-value ≤0.001
	1 vs. 0	1.55 [1.37, 1.75]*	1.53 [1.32, 1.72]*
	2 vs. 0	3.36 [2.84, 3.98]*	2.68 [1.97, 2.89]*
	3 vs. 0	4.91 [4.16, 5.804]*	3.10 [2.54, 3.74]*

* =  logistic regression analysis: OR [95% CI].

Model 1: age, sex.

Model 2, age, sex, SES, partnership, depression according to PHQ-2 ≥3 and anxiety according to GAD-2 ≥3, CHD, MI, Stroke, PAD, Diabetes, Dyslipidemia, Hypertension, Heart Failure, Atrial Fibrillation, and Obesity.

## Discussion

The main findings of our study were that 19% of the participants endorsed clinically significant complaints of sleep disturbances. Increasing severity of complaints of sleep disturbances was associated with an increased occurrence of cardiovascular risk factors and diseases, elevated levels of fibrinogen and CRP. Clinically significant anxiety and depression were most strongly correlated with the severity of self-rated sleep disturbances [1]. After multivariate adjustment robust associations with coronary heart disease, myocardial infarction and dyslipidemia remained. Complaints of sleep disturbances were strong and independent contributors to self-perceived poor physical health beyond depression and anxiety.

The occurrence of 19% clinically significant complaints of sleep disturbances in the sample corresponds to a weighted prevalence rate of 18.5% for the region Mainz-Bingen/Germany. This prevalence and the preponderance of female sex, single persons and lower socioeconomic status is in line with recent reviews [1], [19], [20] and surveys from other regions [21]–[23]. Even though, complaints of sleep disturbances were assessed by a single item, the definition is consistent with other classification approaches [24], [25]. Contrary to our expectations, a correlation of the severity of complaints of sleep disturbances with age was only found for women. It might be speculated that this difference is related to postmenopausal changes in women, which increase the likelihood for sleep disturbances [26].

With respect to biomarkers of inflammation, levels of fibrinogen and CRP increased with the severity of sleep disturbances. However, after multivariable adjustment these associations disappeared. This is in line with a previous large scale cross sectional study of n = 8547 adults from Norway [4], which also found no consistent associations between symptoms of insomnia and CRP. Our findings extend the study of Laugsand et al. (2012) [4] by showing the same inconsistency for fibrinogen. Following Laugsand et al. (2012) [4] we assume it is unlikely that elevated markers of inflammation such as CRP or fibrinogen are crucial factors linking insomnia with coronary heart disease. However, conclusions of this study as well as ours are limited due to reliance on self-reported symptoms of insomnia.

With respect to medical diseases our findings correspond to the previous literature, which described strong positive associations between sleep disturbances and the comorbidity with common medical conditions [1], [3], [25]. In accordance with a corresponding analysis of data from the Behavioral Risk Factor Surveillance System [25], comprising n = 138201 individuals from the USA, we found robust dose-dependent associations of complaints of sleep disturbances with coronary heart disease, myocardial infarction and a measure of metabolic disorders (i.e. in our study dyslipidemia versus obesity in Grandner et al. 2012 [25]). Persons who suffered from sleep disturbances nearly every day as compared to good sleepers had a 60–70% increased likelihood for the occurrence of a medical history of coronary heart disease, myocardial infarction or dyslipidemia. The robust association of sleep disturbances with dyslipidemia is in line with several studies, showing that especially obstructive sleep apnea is associated with dyslipidemia [27], [28]. However, we assume that not only obstructive sleep apnea may account for this association, firstly because sleep apnea is far less prevalent in the community than primary insomnia and secondly because we have controlled for age and obesity, strong correlates of both sleep apnea syndrome and dyslipidemia. We assume that sleep disturbances may influence the lipid metabolism by several pathways. On the one hand, sleep disturbances are accompanied by unhealthy behavior such as stressful overeating, unhealthy diet and physical inactivity, which can contribute to the development of dyslipidemia [29]–[31]. On the other hand, experimental and longitudinal studies suggested that poor sleep may directly increase total cholesterol by alterations in physiological mechanisms of lipid metabolism [5], [28], [30], [32].

Our findings suggest that complaints of sleep disturbances are a very important issue for patients and particularly for patients with cardiovascular disease. Given that self-reported symptoms of insomnia result in an at least moderately increased mortality of cardiovascular patients [33], complaints of sleep disturbances should be a routine focus of concern for general practitioners. The necessity of routinely inquiring for quality of sleep is also supported by the strong and robust association of complaints sleep disturbances with self perceived physical health status [34]: Persons with sleep disturbances nearly every day have a 4.9 fold increased risk to rate their physical status as less good or badly as compared to good sleepers. Even after comprehensive adjustment for cardiovascular risk factors, depression, anxiety and medical disease burden their risk was still 3.8 fold increased.

The main limitation of our study is the use of one single item for determining complaints of sleep disturbances. Due to this limitation inferences about the specific nature of the sleep disturbances, e.g. Obstructive Sleep Apnea Syndrome, Restless Leg syndrome or primary insomnia, are not possible. Further, no objective measures of sleep (e.g. polysomnography) or sleep duration was available. However, previous studies showed that the associations of sleep disturbances with medical diseases were independent from the polysomnographic determination of specific sleep disorders [3]. As the history of CHD, MI and HF were based on the computer-assisted interview, we cannot preclude that this may have influenced the associations reported: Prevalence of CHD or MI might be underestimated due to underdiagnosis, or silent myocardial ischemia. Another important limitation pertains to the cross sectional approach of our study, which precludes any causal inferences about the underlying pathophysiological mechanisms linking sleep disturbances with cardiovascular disease. Previous research demonstrated a bidirectionality of these associations [5]. On the one hand, sleep disturbances are risk factors for the development and course of cardiovascular diseases [33], [35], [36] and mental disorders [1]. On the other hand, sleep disturbances may be directly related to mental disorders [1], psychological consequences of severe medical diseases [37] or their physiological correlates such as sympathetic overactivity in heart failure [38]. However, our study was not designed to elucidate the underlying mechanisms but to determine the size of the problem in a large representative sample of Germany.

In conclusion, there was a high rate of complaints about sleep disturbances in the general population. Complaints of sleep disturbances were strongly and robustly associated with coronary heart disease, myocardial infarction and dyslipidemia. Sleep disturbances had a strong and robust impact on self-perceived health after adjustment for depression, anxiety and medical disease burden. Given the strong impact of poor sleep on self-perceived health and its detrimental health effects increased efforts should be undertaken for the identification and treatment of sleep disturbances [1], [39]. The single question about sleep disturbances from the PHQ depression module constitutes an easy applicable tool for the identification of clinically relevant sleep disturbances. Thus, our findings might stimulate the use of this item in addition to the PHQ screening items for depression and anxiety.
